# Supplementary material for: Content-rich biological network constructed by mining PubMed abstracts
Source: BMC Bioinformatics. 2004 Oct 8;5:147. doi: 10.1186/1471-2105-5-147 (PMC528731; doi:10.1186/1471-2105-5-147)
Supplement: Additional File 2 — The original results of the above study (non-essential files are deleted to keep the file size under the limit set by BMC bioinformatics). [file 1471-2105-5-147-S2.bz2 › chilibotAdditionalFile2/dip50/234ID8006584E758/html/IL2RB_IL2RA.html]

 


 **IL2RB** and **IL2RA** 
  
Found 1183 abstracts in PubMed, retrieved 50.  
 

 What does Google say? 
 PDF only 
| .edu only 

---

**Interactive relationship** (e.g. stimulation, inhibition, etc)

**Stimulatory relationship**- Flow cytometric analysis of the IL 2 receptor  [ **IL2RB** ]  CD25  [ **IL2RA** ]  showed that the normal downregulation kinetics following stimulation induced CD25  [ **IL2RA** ]  upregulation were slowed under 5% O 2, such that the 5% O2 cultures had a greater number of CD25  [ **IL2RA** ]  cells, and those CD25  [ **IL2RA** ]  cells expressed an average n = 6 of 41% higher levels of CD25  [ **IL2RA** ]  receptor per cell.  Ref: 12042052 Cytotherapy, 2000
**Neutral relationship**- In addition, cord blood contained a small population of CD25  [ **IL2RA** ]  cells approximately 2% of CD4 T cells that were CD152 and CD122  [ **IL2RB** ]  low and displayed signs of activation.  Ref: 12047748 Immunology, 2002

**Non-interactive relationship** (e.g. studied together, co-existance, homology, etc.)

- Labeling with CD25  [ **IL2RA** ]  and CD122  [ **IL2RB** ]  antibody was evident on the head and the middle piece in fresh sperm, while in sperm centrifuged on a Percoll gradient a weak labeling was observed only on the principal piece.  Ref: 12230826 Arch Androl< MedlineTA>Arch Androl, 2002
- Parameters of cellular and humoral immunity differential blood count, T cell markers CD2, CD3, CD4, and CD8, B cell markers CD19 and CD20, monocyte markers CD13 and CD14, NK natural killer cell marker CD16, activation markers CD25  [ **IL2RA** ] , CD26, CD69, and HLA DR, and cytokines IL 1 receptor antagonist IL 1RA, IL 2, soluble IL 2 receptor  [ **IL2RB** ]  sIL 2R, IL 6, IL 10, and TGFbeta were measured in venous blood.  Ref: 12074819 Eur Urol, 2002
- Murine studies have suggested that a population of CD4 T cells expressing the alpha chain of the interleukin IL 2 receptor  [ **IL2RB** ]  CD25  [ **IL2RA** ]  are phenotypically anergic in response to T cell receptor stimulation and can suppress the function of CD4 and CD8 T cells.  Ref: 12514432 J Immunother< MedlineTA>J Immunother, 2002
- The interleukin 2 receptor alpha  [ **IL2RA** ]  IL 2Ralpha forms, together with IL 2Rbeta and gammac chains, a high affinity IL 2 receptor  [ **IL2RB** ]  that is important for IL 2 responsiveness and normal T cell function.  Ref: 12231477 Eur Cytokine Netw< MedlineTA>Eur Cytokine Netw, 2002
- The expression of CD25  [ **IL2RA** ]  and CD122  [ **IL2RB** ]  correlated negatively with fresh sperm concentration, but in sperm centrifuged on a Percoll gradient there was no correlation.  Ref: 12230826 Arch Androl< MedlineTA>Arch Androl, 2002
- Twenty sperm samples from oligospermic men were incubated with CD25  [ **IL2RA** ] , a mouse monoclonal antibody specific for IL 2alpha chain receptor, and CD122  [ **IL2RB** ] , a mouse monoclonal antibody specific for IL 2beta chain receptor.  Ref: 12230826 Arch Androl< MedlineTA>Arch Androl, 2002
- Supporting this view were the substantially diminished levels of IL 2, CD25  [ **IL2RA** ] , CD122  [ **IL2RB** ] , and CD132 mRNA found in AGC10 containing cultures of PHA activated lymphocytes.  Ref: 12467977 Mol Biochem Parasitol< MedlineTA>Mol Biochem Parasitol, 2002
- BACKGROUND We have previously demonstrated that the proteolytic activity of Der p 1 selectively cleaves human CD25  [ **IL2RA** ] , the 55 kDa alpha subunit of the IL 2 receptor  [ **IL2RB** ] .  Ref: 11994097 Clin Exp Allergy, 2002
- The OKT3 driven induction of the IL 2 receptor  [ **IL2RB** ]  CD25  [ **IL2RA** ]  and the proliferation of T lymphocytes in response to phorbol myristate acetate were not affected by the addition of RBC.  Ref: 11986282 Clin Diagn Lab Immunol, 2002
- CONCLUSION The monoclonal antibody against IL 2 receptor  [ **IL2RB** ]  alpha chain CD 25  [ **IL2RA** ]  has a marked effect upon severe steroid refractory GVHD.  Ref: 12812665 Zhonghua Yi Xue Za Zhi, 2003
- The inhibitory effect on human T cells was assessed and demonstrated at the level of proliferation DNA synthesis, IL 2 secretion, and IL 2 receptor  [ **IL2RB** ]  CD25  [ **IL2RA** ]  upregulation.  Ref: 12835898 Inflamm Res, 2003
- Prior to testing this strategy in patients, our goal was to develop a clinical scale SD process, which involves co culture of donor lymphocytes and irradiated recipient cells, followed by the addition of an immunotoxin IT directed against the alpha chain of the IL 2 receptor  [ **IL2RB** ]  CD25  [ **IL2RA** ] , expressed on activated donor T cells.  Ref: 12473206 Cytotherapy, 2002
- RESULTS Intra individual monitoring of immune parameters revealed an up regulation of IL 2 receptor  [ **IL2RB** ]  CD25  [ **IL2RA** ]  expression on CD4 and CD8 T cells together with increases in sIL 2R levels in patients with acute rejections.  Ref: 11983203 Clin Chim Acta, 2002
- Whole blood immunophenotyping was performed to study expression of the IL 2 receptor  [ **IL2RB** ]  chains on T lymphocytes and natural killer cells and to further characterize CD4 CD25  [ **IL2RA** ]  T cells.  Ref: 12200381 Blood, 2002
- When we compared mean nocturnal levels, CD16 and CD25  [ **IL2RA** ]  T and B activated lymphocytes with expression of the a chain of IL 2 receptor  [ **IL2RB** ]  levels were higher, while CD8, CD8bright, CD20 total B cells, TcRd1 epitope of the constant domain of d chain of T cell receptor 1 and dTcS1 epitope of the variable domain of d chain of T cell receptor1 levels were lower in cancer patients.  Ref: 12797908 Int J Immunopathol Pharmacol< MedlineTA>Int J Immunopathol Pharmacol, 2002
- Activation markers CD25  [ **IL2RA** ] , CD122  [ **IL2RB** ] , CD69 and CD80 were also present on fetal T cells.  Ref: 12880639 Dev Comp Immunol, 2003
- BACKGROUND Basiliximab is a chimeric human mouse monoclonal antibody directed against the alpha chain of the IL 2 receptor  [ **IL2RB** ] , CD25  [ **IL2RA** ] , which has been reported as successfully reducing rejection in adult renal transplant recipients.  Ref: 12105256 Nephrol Dial Transplant, 2002
- Cell surface expression of CD25  [ **IL2RA** ] , CD122  [ **IL2RB** ] , CD132, and CD69 were also determined and no statistical differences were found between both groups.  Ref: 12635575 Allergy Asthma Proc< MedlineTA>Allergy Asthma Proc, 2002
- However, eosinophils with sparse granulation, positivity for CD25  [ **IL2RA** ] , elevated serum levels of soluble IL 2 receptor  [ **IL2RB** ] , and elevated serum levels of eosinophil cationic protein suggested activation of eosinophils.  Ref: 12462027 Rinsho Ketsueki, 2002
- The strong initial fluorescence intensity and, subsequently, a labeling index yielded by CD25  [ **IL2RA** ]  and CD122  [ **IL2RB** ]  decreased in sperm centrifuged on a Percoll gradient p <.  Ref: 12230826 Arch Androl< MedlineTA>Arch Androl, 2002
- The suppressive activity of these cells was contact dependent and associated with the lack of IL 2 receptor  [ **IL2RB** ]  IL 2R alpha chain CD25  [ **IL2RA** ]  expression in target cells.  Ref: 12163566 J Exp Med, 2002
- IL 2 was not detected in cell culture supernatants, but IL 2 receptor  [ **IL2RB** ]  alpha chain CD25  [ **IL2RA** ]  was detected in low density on a small proportion of T cells activated by C1q bearing immune complexes.  Ref: 12519387 Clin Exp Immunol, 2003
- BACKGROUNDTo define whether immunosuppressive agents that block the interleukin IL 2 pathway could prevent activation induced cell death of activated T cells in the graft, we measured expression of IL 2, IL 2 receptor  [ **IL2RB** ]  alpha chain CD25  [ **IL2RA** ] , IL 15, Fas, and Fas ligand by real time reverse transcription polymerase chain reaction in cardiac allografts.METHODSWe characterized the phenotype of the infiltrating cells CD3, CD68, CD25  [ **IL2RA** ]  by immunohistochemistry.  Ref: 12777860 Transplantation, 2003
- We investigated a possible relation between prolactin receptor PRL R or IL 2 receptor  [ **IL2RB** ]  alpha IL 2Ralpha, CD25  [ **IL2RA** ]  expression on circulating T lymphocytes and their apoptosis in patients with breast cancer.  Ref: 12698200 Br J Cancer, 2003
- We found a defective T cell response in malnourished children, as indicated by diminished levels of circulating total CD3, helper CD4, IL 2 receptor  [ **IL2RB** ]  bearing CD4 CD25  [ **IL2RA** ]  and memory helper T cell responses CD4 CD45RO in keeping with the decreased specific IgE levels against Ascaris lumbricoides.  Ref: 12848201 J Trop Pediatr, 2003
- Cells stained with Annexin V Anx and antibodies to CD56, CD3, CD95, CD25  [ **IL2RA** ] , CD122  [ **IL2RB** ]  or CD132 were examined by flow cytometry.  Ref: 12594840 Eur J Immunol, 2003
- Increased CD25  [ **IL2RA** ]  expression, especially in CD4 T cells but also in CD8 T cells, without increases in expression of the beta and gamma chains of the IL 2 receptor  [ **IL2RB** ]  was detected in the IL 2 group.  Ref: 12200381 Blood, 2002
- Most of the surface membrane antigens CD5, CD11c, CD19, CD20, CD21, CD22, CD23, CD25  [ **IL2RA** ] , CD40, CD45, VD79b, CD80, CD95, CD122  [ **IL2RB** ] , CD124, CD126, CD130, CD154, IgM, and IgD showed a similar expression pattern in both UM CLL and M CLL patients.  Ref: 12631259 Eur J Haematol, 2003
- We observed that eosinophils from most patients with hypereosinophilia express the alpha chain of the IL 2 receptor  [ **IL2RB** ]  CD25  [ **IL2RA** ] ,.  Ref: 12672048 Eur J Immunol, 2003
- Parameters of cellular and humoral immunity differential blood count, T cell markers CD2, CD3, CD4, and CD8, B cell markers CD19 and CD20, monocyte markers CD13 and CD14, NK cell marker CD16, activation markers CD25  [ **IL2RA** ] , CD26, CD69 and HLA DR, and cytokines IL 1 receptor antagonist IL 1RA, IL 2, soluble IL 2 receptor  [ **IL2RB** ]  sIL 2R, IL 6, IL 10, and TGFbeta were measured in peripheral venous blood.  Ref: 12779014 Folia Biol Praha , 2003
- Induction of interleukin 2 receptor alpha  [ **IL2RA** ]  IL 2Ralpha expression by interleukin 2 important role of the interleukin 2 receptor  [ **IL2RA** ]  beta chain  [ **IL2RB** ]  region between the two Stat5 docking sites.  Ref: 12231477 Eur Cytokine Netw< MedlineTA>Eur Cytokine Netw, 2003
- Like living Trypanosoma cruzi, its AGC10 membrane glycoprotein inhibits interleukin 2 IL 2 secretion and membrane expression of CD25  [ **IL2RA** ] , CD122  [ **IL2RB** ] , and CD132 the components of the high affinity IL 2 receptor  [ **IL2RB** ]  by activated human lymphocytes.  Ref: 12467977 Mol Biochem Parasitol< MedlineTA>Mol Biochem Parasitol, 2003
- There is a dynamic pre association of CD25  [ **IL2RA** ]  and CD122  [ **IL2RB** ]  leading to the loose association of the heterodimer with membrane microdomains.  Ref: 12121224 Immunol Cell Biol, 2002
- Most investigated Treg cells are CD4 pos cells expressing the alpha chain of the IL 2 receptor  [ **IL2RB** ]  CD25  [ **IL2RA** ] .  Ref: 12848995 Autoimmun Rev, 2002
- CD25  [ **IL2RA** ] , CD152 and CD122  [ **IL2RB** ]  cells were present in paediatric thymus 10% of CD4 CD8 thymocytes expressing signs of recent selection CD69 and in cord blood 5% of CD4 cells where they showed a naive phenotype.  Ref: 12047748 Immunology, 2002
- In addition to the activation memory T cell antigens mentioned above they also expressed intracellular CD152 CTLA 4 as well as enhanced levels of cell surface CD122  [ **IL2RB** ] , similar to the murine CD4 CD25  [ **IL2RA** ]  regulatory counterpart.  Ref: 12047748 Immunology, 2002
- AGC10 also decreased the intracellular levels of CD25  [ **IL2RA** ] , CD122  [ **IL2RB** ] , and CD132 in CD4 and CD8 cells stimulated with the T cell mitogen phytohemagglutinin PHA.  Ref: 12467977 Mol Biochem Parasitol< MedlineTA>Mol Biochem Parasitol, 2002
